# Thalassemia and Hemoglobin E in Southern Thai Blood Donors

**DOI:** 10.1155/2014/932306

**Published:** 2014-06-23

**Authors:** Manit Nuinoon, Kwanta Kruachan, Warachaya Sengking, Dararat Horpet, Ubol Sungyuan

**Affiliations:** ^1^School of Allied Health Sciences and Public Health, Walailak University, Nakhon Si Thammarat 80161, Thailand; ^2^Regional Blood Centre XI, National Blood Centre Thai Red Cross Society, Nakhon Si Thammarat 80110, Thailand; ^3^Center for Scientific and Technological Equipment, Walailak University, Nakhon Si Thammarat 80161, Thailand

## Abstract

Thalassemia and hemoglobin E (Hb E) are common in Thailand. Individuals with thalassemia trait usually have a normal hemoglobin concentration or mild anemia. Therefore, thalassemic individuals who have minimum acceptable Hb level may be accepted as blood donors. This study was aimed at determining the frequency of *α*-thalassemia 1 trait, *β*-thalassemia trait, and Hb E-related syndromes in Southern Thai blood donors. One hundred and sixteen voluntary blood donors, Southern Thailand origin, were recruited for thalassemia and Hb E screening by red blood cell indices/dichlorophenolindophenol precipitation test. *β*-Thalassemia and Hb E were then identified by high performance liquid chromatography and 4 common *α*-thalassemia deletions were characterized by a single tube-multiplex gap-polymerase chain reaction. Overall frequency of hemoglobinopathies was 12.9%, classified as follows: homozygous *α*-thalassemia 2 (1.7%), heterozygous *α*-thalassemia 1 (1.7%), heterozygous *β*-thalassemia without *α*-thalassemia (0.9%), heterozygous Hb E without *α*-thalassemia (5.2%), double heterozygotes for Hb E/*α*-thalassemia 1 (1.7%), homozygous Hb E without *α*-thalassemia (0.9%), and homozygous Hb E with heterozygous *α*-thalassemia 2 (0.9%). The usefulness of thalassemia screening is not only for receiving highly effective red blood cells in the recipients but also for encouraging the control and prevention program of thalassemia in blood donors.

## 1. Introduction

*α*-Thalassemia, *β*-thalassemia, and Hb E (*β* ^codon 26, Glu→Lys^), the most common genetic blood disorders, are considered not only public health problems but also socioeconomic problem in Thailand [1, 2]. The frequencies of *α*-thalassemia, *β*-thalassemia, and Hb E carriers in Thailand were ranged from 20 to 30%, 3 to 9% and 10 to 60%, respectively, and vary from region to region [3, 4]. These abnormal globin genes in different combinations lead to more than 60 thalassemia syndromes including three severe thalassemic diseases found in Thailand such as Hb Bart's hydrops fetalis (homozygous *α*-thalassemia 1, –/–), homozygous *β*-thalassemia (*β* ^+^/*β* ^+^, *β* ^+^/*β* ^0^, or *β* ^0^/*β* ^0^), and *β*-thalassemia/Hb E (*β* ^+^/*β* ^E^ or *β* ^0^/*β* ^E^). Thai married couples are at risk of giving birth to babies with severe hemoglobinopathies about 5.6% [2]. To reduce the number of affected patients with severe thalassemia syndrome, the prevention and control program for thalassemia in Thailand is necessary by screening the carriers of abnormal genes in general population [5]. Concerning the precision of thalassemia diagnosis, the red blood cells from Hb E donors (Hb E trait or homozygous Hb E) can cause the misdiagnosis of thalassemia in the normal recipients (false positive) or the red blood cells from normal donors can cause the misdiagnosis of thalassemia in the thalassemic recipients (false negative) [6]. The prevalence of thalassemia and abnormal hemoglobin in general population has been reported in several studies. In Thailand the hemoglobin concentration of thalassemia carriers is variable ranging from anemia to normal range because there are different numbers of globin gene defects and interactions of *α*- and *β*-thalassemia in a region with high frequency [7, 8]. According to AABB's (American Association of Blood Banks) Technical Manual, minimal hemoglobin concentration for accepting a blood donor is not less than 125 g/L for allogeneic donor and 110 g/L for autologous donor [9]. Therefore, the thalassemia traits with normal hemoglobin concentration could donate their blood. Nowadays, the study of thalassemia and abnormal hemoglobin in blood donors has been reported in several populations with different frequencies [10–15]. This report is the first published data in Thailand that provides the useful data for hemoglobinopathies among blood donors for reducing a number of severe thalassemia patients.

## 2. Materials and Methods

A cross-sectional study was conducted at Regional Blood Centre XI, National Blood Centre Thai Red Cross Society, Nakhon Si Thammarat from July to September 2013. The samples were collected from different hospitals located in Southern Thailand. The study protocol was reviewed and approved by the institutional review board of Walailak University, Thailand (IRB number 2013-011). Informed consent was confirmed by the IRB. After informed consent was obtained, peripheral blood samples anticoagulated with EDTA were collected from 116 Southern Thai voluntary blood donors who had passed the donor self-exclusion according to AABB criteria. Briefly, donors must be between the ages of 17 and 70 years and must weigh more than 45 kilograms and be in good health with no risks of infectious diseases. According to blood donor criteria of Thai Red Cross Society, minimal Hb concentration for accepting a blood donor is not less than 120 g/L and 130 g/L for female and male, respectively. Hb concentration was estimated by copper sulfate (CuSO_4_) specific gravity method. Copper sulfate with specific gravity of 1.052 (representing Hb concentration of 120 g/L) was used for Hb screening in female donors and specific gravity of 1.053 (representing Hb concentration of 130 g/L) was used for Hb screening in female donors. The individuals who have a drop of blood floats or takes too long to sink are deferred and were excluded from this study.

Complete blood count (CBC) was determined by using an automated blood cell analyzer, MEK-8222 K (NIHON KOHDEN, Tokyo, Japan). Mean corpuscular volume (MCV) and dichlorophenolindophenol precipitation test by using the KKU-DCIP Clear Reagent Kit (PCL Holding, Bangkok, Thailand) were used as thalassemia and Hb E screening methods, respectively. The positive results of MCV (<80 fL) and/or DCIP were subsequently performed Hb typing by high performance liquid chromatography (HPLC). Hemoglobin type and quantitation of Hb A_2_/E, Hb A, and Hb F were conducted by an automated hemoglobin cation exchange HPLC (Variant *β*-thalassemia short program, Bio-rad Laboratories, Hercules, CA).

Genomic DNA was extracted from peripheral blood leukocytes by using the Genomic DNA Extraction Kit (Geneaid, Taipei, Taiwan) according to the manufacturer's instructions. Concentration and quality of a sample of genomic DNA are measured with ND-1000 (NanoDrop Technologies, Wilmington, DE). To characterize the *α*-globin gene deletions, the 3.7 kb (-*α* ^3.7^) and 4.2 kb (-*α* ^4.2^) deletion types for *α*-thalassemia 2, Southeast Asian (–^SEA^), and THAI (–^THAI^) deletions' types for *α*-thalassemia 1 were performed by multiplex GAP-PCR [16] in the all samples with low MCV (≤80 fL). Therefore, *α*-thalassemia 2 trait was not separated from normal individuals by DNA testing in this study. PCR products were amplified by GeneAmp PCR system 9700 (Perkin Elmer, CT, USA). 8 *μ*L of PCR products was electrophoresized in 1.5% agarose gel, stained with ethidium bromide, and visualized and photographed by a gel documentation system (G-Box, SynGene, Frederick, MD, USA).

### 2.1. Statistical Analysis

The data were presented as mean ± standard deviation (SD). Statistical comparison of hematological data was conducted with the nonparametric Kruskal-Wallis test using SPSS version 17.0 (SPSS, Chicago, IL, USA).* P* values < 0.05 were considered statistically significant.

## 3. Results

A total of 116 voluntary blood donors were recruited in this study, 65 males (56%) and 51 females (44%). All voluntary blood donors lived in Southern Thailand. The mean age (± SD) was 33 ± 11.3 years (range: 17–58 years) old. The most common status of the subjects was single (55%). Blood groups “O” and “B” were found to be the codominant and all donors were positive Rh (D) blood group. The average values of all red cell parameters were ranged in the normal values. The high variation of red cell volume (MCV) was observed (SD = 6.94) because of hemoglobinopathies as shown in Table 1.  Table 2 represents the prevalence of hemoglobinopathies in 116 Southern Thai blood donors. According to our study design, *α*-thalassemia 1, *β*-thalassemia, and Hb E were focused on and identified because they can cause the severe thalassemia in the next generation such as Hb Bart's hydrops fetalis, homozygous *β*-thalassemia, and *β*-thalassemia/Hb E disease. The normal individuals and *α*-thalassemia 2 traits were not differentiated by DNA analysis because of its high cost and its low importance. Out of 116 donors, 101 (87.1%) donors were diagnosed as normal or heterozygous *α*-thalassemia 2 and 15 (12.9%) donors were interpreted as thalassemia and/or abnormal hemoglobin. Among these hemoglobinopathies, Hb E-related disorders (both Hb E heterozygote and homozygote with and without *α*-thalassemia) are the most common form of hemoglobinopathies accounting for 8.6% (10/116), followed by heterozygous *α*-thalassemia 1 (both heterozygous *α*-thalassemia 1 [1.7%] and double heterozygote for Hb E and heterozygous *α*-thalassemia 1 [1.7%]) accounting for 3.4% (4/116) and heterozygous *β*-thalassemia accounting for 0.9% (1/116).

**Figure 1:**
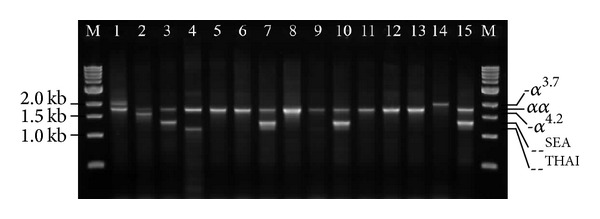
Representative 1.5% agarose gel electrophoresis of the amplified PCR products for characterizing *α*-globin gene deletion types by multiplex gap PCR. The 2 kb, 1.8 kb, 1.6 kb, 1.4 kb and 1.2 kb represent 3.7 kb deletion fragment (-*α* ^3.7^), normal fragment (*αα*), 4.2 kb deletion fragment (-*α* ^4.2^), SEA type deletion fragment (–^SEA^), and THAI type deletion fragment (–^THAI^), respectively. The M represents the 1 kb DNA ladder. Lanes 1–4 (positive controls) are genotypes as follows: -*α* ^3.7^/*α* *α*, -*α* ^4.2^/*α* *α*, –^SEA^/*αα*, and –^THAI^/*αα*, respectively. Lanes 5, 6, 8, 9, and 11–13 are normal *α*-globin genotype (*αα*/*αα*). Lanes 7, 10, and 15 are heterozygous *α*-thalassemia 1 (–^SEA^/*α* *α*). Lane 14 is homozygous *α*-thalassemia 2 (-*α* ^3.7^/-*α* ^3.7^).

We divided the 116 Southern Thai blood donors into seven groups according to the type of thalassemia and Hb E (red cell indices, Hb type, and DNA analysis were used to interpret the phenotype). Hematological findings are listed and compared as shown in Table 3. Multiplex gap PCR for determining *α*-globin gene deletions as depicted in Figure 1. Normal and *α*-thalassemia 2 traits are the major component of the studied donors. We found statistically significant differences for all hematological parameters among the four groups (groups I, II, IV, and V); when they were analyzed using the nonparametric Kruskal-Wallis test, MCV, MCH, and % Hb A_2_/E were most obvious (*P* < 0.001). In this study, homozygous Hb E without *α*-thalassemia interaction had the lowest MCV and MCH compared with other groups. Increased Hb A_2_/E levels were observed in *β*-thalassemia trait, Hb E trait, and Hb E homozygote. Coinheritance of *α*-thalassemia in Hb E heterozygote was found to be decreased hematological parameters compared with Hb E heterozygote with no *α*-thalassemia interaction. In contrast, interaction of *α*-thalassemia in Hb E homozygote was found to have improved hematological parameters (increased Hb, MCV, and MCH) compared with Hb E homozygote with no *α*-thalassemia. Microcytic (MCV < 80 fL) and/or hypochromic (MCH < 27 pg) red blood cells were 25.9% (30/116) and 26.7% (31/116), respectively. Among 30 blood donors with microcytic red blood cells, fifteen blood donors (50%) were found to have hemoglobinopathies and the left blood donors may be having an iron deficiency and/or *α*-thalassemia 2 trait.

HbA_2_/E levels are used to diagnose the *β*-thalassemia trait (%HbA_2_ = 4.0–8.0) and Hb E-related disorders (HbA_2_/E > 10.0%). However, *α*-thalassemia and interaction of *α*-thalassemia in *β*-thalassemia and Hb E-related disorders, red blood cell indices, and Hb typing could not be used for interpretation. In this study, multiplex gap PCR was used to characterize the four common *α*-globin gene deletions (both *α*-thalassemia 1 allele [–^SEA^ and –^THAI^] and *α*-thalassemia 2 allele [-*α* ^3.7^ and -*α* ^4.2^]) as shown in Figure 1.  Table 4 demonstrates the number of risk alleles among thalassemia or Hb E-related blood donors. From all blood donors, there are 17 alleles (from 13 blood donors) that can cause the severe thalassemia in the offspring (risk allele frequency = 3.7%). Among 17 risk alleles, Hb E allele (*β* ^E^) is the most common form of all risk alleles (–/, *β* ^0/+^ and *β* ^E^), 12/17 (70.6%), followed by *α*-thalassemia 1 allele (–/), 4/17 (23.5%), and *β*-thalassemia allele (*β* ^0/+^), 1/17 (5.9%).

## 4. Discussion

Blood donor selection is crucial to ensure the safety of both donors and recipients. According to the standards of the American Association of Blood Banks (AABB), hemoglobin concentration more than 125 g/L was accepted for blood donation [9]. The prevalence of thalassemia and abnormal hemoglobin varies from region to region, the frequency of *α*-thalassemia in Bangkok and northern Thailand was ranging from 20 to 30%, and *β*-thalassemia varies between 3 and 9%. Among abnormal hemoglobin, Hb E is the most common, especially in the northeastern part of Thailand and the junction of Thailand with Laos and Cambodia where its prevalence can reach 50–60% [3, 4, 17]. The prevalence of *β*-thalassemia trait, Hb E trait, homozygous Hb E, and *α*-thalassemia 1 trait in Southern Thai couples was 2.22%, 12.08%, 1.11%, and 3.06%, respectively, and among Thai population; Southern Thai population was found to have the lowest prevalence of thalassemia and Hb E [18]. In this study similar pattern with lower frequency of thalassemia and Hb E was observed in blood donors because Hb concentration in thalassemia carriers (*α*-thalassemia 1 trait, *β*-thalassemia trait, and Hb E-related syndromes) varies ranging from normal value to very slight anemia [7, 19, 20]. Therefore, thalassemic individuals could or could not donate the blood and some thalassemic individuals who have anemia were excluded from this study. The frequencies of thalassemia in blood donors have been reported in several populations [10–12, 21]. For example, among 80 Malaysian blood donors, the frequency of thalassemia was 16.25% which is slightly higher than this study [12]. Tiwari and Chandola [22] reported that the prevalence of microcytosis in Indian blood donors was 5.4% (50/925). Alabdulaali et al. [23] published that sickle cell trait was found 2% (23/1,150) in King Khalid University Hospital (KKUH) in Riyadh. In addition, Bryant et al. [24] found that 2.8% (33/1,162) of the apheresis donors had low mean corpuscular volume values (MCV < 80 fL). In the present study, microcytosis was found to be 25.9% in blood donors. These blood donors could be having hemoglobinopathies and/or iron deficiency [25, 26]. For other red blood cell disorders, glucose-6-phosphate dehydrogenase deficiency was found to be 1.1% (33/3,004) in Italian blood donors [27], 0.3% (1/301) in a metropolitan transfusion service [28], and 0.78% (9/1,150) in King Khalid University Hospital (KKUH) in Riyadh. The importance of glucose-6-phosphate dehydrogenase deficiency is red blood cell destruction in response to several oxidative stresses [29]. Increased osmotic fragility of erythrocytes in 1,464 healthy German blood donors was 1.1% (16/1,464) [30]. Iron deficiency was also observed in blood donors [31, 32]. Donor selection is very important to protect both the donor and the recipient. In this study, two important issues are concerned and highlighted. Firstly, concerning the quality of red blood cells, it is common practice in many hospitals or transfusion service centers to accept blood for transfusion from donors with thalassemia minor. However, two donors with homozygous Hb E from this study have normal Hb levels (12.7 and 13.9 g/dL) with very low MCV (61.6 and 67.6 fL) and MCH (19.7 and 22.0 pg) consistent with the blood smear showing microcytic and hypochromic red blood cells. High quality of packed red cell (PRC) for regularly transfused patients such as severe *β*-thalassemia patients should be considered. The red blood cells from blood donors can cause the misdiagnosis of thalassemia in the recipients (false positive or false negative), for example, individuals who have received blood transfusions from Hb E-related donors (Hb E trait or Hb E homozygote) or Hb E-related individuals who have received transfusions from normal blood donors [6]. Therefore, not only quantitative screening but also qualitative evaluation is necessary for selecting blood for severe thalassemia patients. Secondly, blood donors may be carriers of the hemoglobinopathies without being aware of it because they can donate the blood. In Thailand prevention and control program of severe thalassemia has been established [32]. Screening of hemoglobinopathies in blood donors is one of strategies for prevention and control of severe thalassemia. Hemoglobin Bart's hydrops fetalis, homozygous *β*-thalassemia, and *β*-thalassemia/Hb E are concerned and programmed for prenatal diagnosis in Thailand [33, 34]. Three important risk alleles (*α*-thalassemia 1, *β*-thalassemia, and Hb E) are found in these blood donors and they are at risk of giving birth to babies with severe hemoglobinopathies. Thus, it is very important to characterize the type of hemoglobinopathies and understand the multiple gene-gene interactions in order to provide proper counseling to the blood donors. Furthermore, DNA testing is also necessary to confirm the phenotypes of hemoglobinopathies.

## 5. Conclusion

This preliminary study demonstrated a significant frequency of *α*-thalassemia 1 trait, *β*-thalassemia trait, and Hb E-related disorders in Southern Thai blood donors and revealed similar pattern with lower frequencies in general population because of exclusion criteria of blood donors. To provide the safety of both blood donors and recipients, the screening of thalassemia and Hb E in blood donors was recommended in highly prevalent countries. Both quantitative and qualitative measurements of red blood cells were suggested before transfusing to the patients with red blood cell disorders such as regularly transfused *β*-thalassemia patients. The data obtained from this study also provide useful information for the prevention and control program of thalassemia in Southern Thai blood donors. Additional samples are required to support the importance of the hemoglobinopathy screening and to validate the prevalence of thalassemia and Hb E in blood donors.

## Figures and Tables

**Table 1:** Descriptive data and hematological findings of voluntary blood donors (*n* = 116).

Donor characteristics	Number of donors (%)
Gender	
Male	65 (56)
Female	51 (44)
Status	
Single	64 (55)
Married	47 (41)
Others	5 (4)
ABO blood group system	
A	29 (25)
B	41 (35)
O	38 (33)
AB	8 (7)
Rh (D) blood group system	
Positive	116 (100)
Negative	0 (0)
Age (years)∗	33 ± 11.3 (17–58)
Hematological data∗∗	
RBC (10^12^/L)	5.0 ± 0.60
Hemoglobin (g/L)	140 ± 13.3
HCT (L/L)	0.41 ± 0.04
MCV (fL)	82.8 ± 6.94
MCH (pg)	28.2 ± 2.71
MCHC (g/dL)	34.0 ± 0.69
RDW (%)	12.5 ± 1.16

*Age is presented as mean ± standard deviation (range).

**Hematological data are expressed as mean ± standard deviation.

**Table 2:** Prevalence of thalassemia and Hb E among Southern Thai blood donors.

Diagnosis	Number of donors (%)
Normal and heterozygous *α*-thalassemia 2	101 (87.1)
Homozygous *α*-thalassemia 2	2 (1.7)
Heterozygous *α*-thalassemia 1	2 (1.7)
Heterozygous *β*-thalassemia without *α*-thalassemia	1 (0.9)
Heterozygous Hb E without *α*-thalassemia	6 (5.2)
Double heterozygotes for Hb E/*α*-thalassemia 1	2 (1.7)
Homozygous Hb E without *α*-thalassemia	1 (0.9)
Homozygous Hb E with heterozygous *α*-thalassemia 2	1 (0.9)

Total	116 (100)

**Table 3:** Hematological findings of normal and different types of thalassemia and Hb E.

Group (*n*)	Hb (g/L)	MCV (fL)	MCH (pg)	Hb A_2_/E (%)
I. Normal and heterozygous *α*-thalassemia 2 (*n* = 101)	141 ± 13.4	84.9 ± 4.31	30.0 ± 1.68	—
II. Homozygous *α*-thalassemia 2 and heterozygous *α*-thalassemia 1 (*n* = 4)	129 ± 10.5	65.1 ± 2.44	21.4 ± 0.90	2.9 ± 0.25
III. Heterozygous *β*-thalassemia without *α*-thalassemia (*n* = 1)	139	62.2	20.0	5.0
IV. Heterozygous Hb E without *α*-thalassemia (*n* = 6)	134 ± 9.6	75.1 ± 3.39	25.2 ± 1.16	26.2 ± 0.58
V. Double heterozygotes for Hb E/*α*-thalassemia 1 (*n* = 2)	123 ± 6.4	67.5 ± 1.84	21.8 ± 0.57	19.3 ± 0.64
VI. Homozygous Hb E without *α*-thalassemia (*n* = 1)	127	61.6	19.7	74.5
VII. Homozygous Hb E with heterozygous *α*-thalassemia 2 (*n* = 1)	139	67.6	22.0	75.4
*P* value∗	0.05	<0.001	<0.001	<0.001

Hematological data are expressed either as mean ± standard deviation (SD) or raw data where appropriate (*n* = 1, groups III, VI, and VII).

Hb: hemoglobin; g/L: gram per liter; MCV: mean corpuscular volume; fL: femtoliter; MCH: mean corpuscular hemoglobin; pg: picogram.

*P value was calculated by using the nonparametric Kruskal-Wallis test (groups I, II, IV, and V were compared).

**Table 4:** Number of risk alleles and risk allele frequency in Southern Thai blood donors.

Diagnosis	*α*-Globin and *β*-globin genotypes	Number of alleles∗	Number of risk alleles (risk allele)
Normal, heterozygous *α*-thalassemia 2, and homozygous *α*-thalassemia 2 (*n* = 103)	*αα*/*αα*, −*α*/*αα*, −*α*/−*α*, and *β* ^A^/*β* ^A^	412	0
Heterozygous *α*-thalassemia 1 (*n* = 2)	- -^SEA^/*αα*, *β* ^A^/*β* ^A^	8	2 (- -^SEA^)
Heterozygous *β*-thalassemia without *α*- thalassemia (*n* = 1)	*αα*/*αα*, *β* ^0/+^/*β* ^A^	4	1 (*β* ^0/+^)
Heterozygous Hb E without *α*-thalassemia (*n* = 6)	*αα*/*αα*, *β* ^E^/*β* ^A^	24	6 (*β* ^E^)
Double heterozygotes for Hb E/*α*- thalassemia 1 (*n* = 2)	- -^SEA^/*αα*, *β* ^E^/*β* ^A^	8	4 (2 ∗ *β* ^E^ and 2 ∗ - -^SEA^)
Homozygous Hb E with and without heterozygous *α*-thalassemia 2 (*n* = 2)	−*α*/*αα*, *αα*/*αα*, and *β* ^E^/*β* ^E^	8	4 (*β* ^E^)

Total (*n* = 116)		464	17
Risk allele frequency = 17/464 ∗ 100 = 3.7%			

- -^SEA^: *α*-thalassemia 1 allele with Southeast Asian type deletion; *β* ^0/+^: *β* ^0^ or *β* ^+^-thalassemia allele with uncharacterized *β*-globin gene mutation; *β* ^A^: normal *β*-globin gene; *β* ^E^: Hb E allele.

*The number of alleles was calculated from two alleles of *α*-globin genotype (*αα*/*αα*) and two alleles from *β*-globin genotype (*β*/*β*) [4 alleles were considered per one donor].
